# Receptor autoimmunity: diagnostic and therapeutic implications

**DOI:** 10.1186/s13317-019-0125-5

**Published:** 2020-01-07

**Authors:** Renato Tozzoli

**Affiliations:** Laboratory of Clinical Pathology, S. Maria degli Angeli Hospital, and Consultant Endocrinologist, San Giorgio Clinics, Pordenone, Italy

**Keywords:** Cell-surface receptors, Graves’ disease, Myasthenia gravis, Autoimmune acute encephalitis, Idiopathic membranous nephropathy, Receptor autoantibodies, Immunoassays

## Abstract

Receptor autoimmunity is one of the ways in which autoimmune diseases appear in humans. Graves’ disease, myasthenia gravis, idiopathic membranous nephropathy, and autoimmune acute encephalitis are the major autoimmune diseases belonging to this particular group. Receptor autoimmune disease are dependent on the presence of autoantibodies directed against cell-surface antigens, namely TSH receptor in thyrocytes, acetylcholine receptor in neuromuscular junction, phospholipase 2 receptor in podocytes, and NMDA receptor in cortical neurons. In this article we outline the distinctive features of receptor autoimmunity and the specific relationship between the autoimmunology laboratory and the presence/concentration of autoantibodies. Some immunological features distinguish receptor autoimmunity. Anti-receptor autoantibody pathologies are considered T cell-dependent, B-cell-mediated autoimmune disorders: the knowledge about the presence of circulating and/or localized autoantibodies to target organs and identification of autoantigens involved in the autoimmune reaction is of paramount importance. Due to the close correlation between the concentration of anti-receptor autoantibodies, the autoimmune target of some cell-surface receptors and the intensity of symptoms, the measurement of these immunoglobulins has become central to diagnose autoimmune diseases in all affected patients, not just in clinically dubious cases. The measurement of autoantibodies is also relevant for differential diagnosis of autoimmune and non-autoimmune forms with similar symptoms. From the methodological point of view, quantitative immunoassay methods of measurement should be preferred over semi-quantitative ones, for the capacity of the first class of methods to define precisely the reference ranges and decision levels overcoming the measurement uncertainty of semi-quantitative methods.

## Introduction

Autoimmunity against cell-surface receptors represents a field of significant interest in autoimmune diagnostics, due to the unique characteristics of syndromes and human pathologies that have over time seen recognized cell-surface molecules as target of immune reactions.

The term ‘receptor autoimmunity’ was coined by Duncan D. Adams, a New Zealand endocrinologist, who in the mid-1950s-highlighted the pathogenic role of autoantibodies against the TSH receptor (TSHR), at the time known as LATS (long-acting thyroid stimulator), in autoimmune hyperthyroidism or Graves’ disease (GD) [1, 2].

GD is the prototypic example of autoimmune pathology, in which the diagnostic and pathogenic direct effect of functional autoantibodies against TSHR (TRAbs) has been demonstrated, both in the case of stimulating and blocking immunoglobulins; TRAbs with opposite effects may be present during the course of the disease and determine the symptoms, according to Roitt’s type V and VI immunopathogenic mechanisms.

Over the years other autoimmune diseases have been shown to recognize a similar pathogenetic pathway. As early as 1960, the hypothesis of the role of antibodies against the acetylcholine receptor (AChR) in the pathogenesis of myasthenia gravis (MG) was assumed and then confirmed [3, 4]. In this autoimmune pathology, anti-AChR autoantibodies (AChRAb) play mainly a blocking role, are directed against extracellular epitopes of AChR and inhibit neuromuscular transmission (type II and VI immunopathogenic mechanisms) [5].

The same mechanisms involving autoantibodies directed against the N-methyl-d-aspartate receptor (NMDAR) is responsible for the clinical picture of autoimmune acute encephalitis (anti-NMDAR encephalitis) [6, 7].

Likewise, an important kidney disease finds its etiopathogenesis in the presence of antibodies against the phospholipase receptor A2 (PLA2R): it is the case of idiopathic membranous nephropathy (IMN), for which it was only recently possible to clarify the role of some receptor autoantibodies (type VI immunopathogenic mechanism) [8, 9].

In other autoimmune systemic and organ-specific diseases (systemic sclerosis, rare forms of diabetes, and dilated cardiomyopathy) the significance of the receptor autoimmunity was recently clarified.

Among these pathologies, GD presents a very high prevalence/incidence in humans, compared to other rare or less frequent autoimmune receptor diseases, in particular myasthenia gravis, autoimmune encephalitis, and membranous nephropathy.

In this article we outline the distinctive features of receptor autoimmunity and the specific relationship between the autoimmunology laboratory and the receptor autoimmunity, we present the main autoimmune diseases, on which an involvement of an autoimmune attack against receptor antigens is demonstrated, and finally we show the most recent knowledge on the therapeutic role of receptor peptides in clinical management of these diseases.

### Distinguishing receptor autoimmunity

Some immunological features distinguish receptor autoimmunity: anti-receptor autoantibody pathologies are considered T-cell-dependent, B-cell-mediated autoimmune disorders [10]. In these diseases, the knowledge about the presence of circulating and/or localized autoantibodies to target organs and identification of autoantigens involved in the autoimmune reaction is of paramount importance.

In fact, these specific immunoglobulins act directly stimulating or blocking the target receptor and consequently determining the specific symptoms of the pathologies at stake. Table 1 shows the main properties of antigens involved in receptor autoimmunity, and Table 2 describes the functional autoantibodies responsible for the symptoms. These aspects are of great interest to the laboratory medicine, because measuring circulating concentrations of these pathogenic immunoglobulins is crucial for diagnosis and therapeutic monitoring.

**Table 1:** The main autoantigens in receptor autoimmunity

Autoantigen	Achronym	Molecular weight (kDa)	Domains
TSH receptor	TSHR	84.5	A and B subunits
Acetylcholine receptor	AChR	250.0	α, β, γ, δ, and ε subunits
N-methyl-d-aspartate receptor	NMDAR	710.0	ATD, ABD, TMD, CTD
Phospholipase 2 receptor	PLAR2	180.0	ECD, TMD, CTD

*ECD* extracellular domain, *ABD* agonist binding domain, *TMD* transmembrane domain, *CTD* intracellular domain

**Table 2:** The main autoantibodies in receptor autoimmune diseases and their pathogenic actions

Autoantibody	Acronym	Subclasses	Action
TSH receptor antibodies	TRAb	IgG	Stimulating TSH receptor Blocking TSH receptor Apoptosis of thyrocyte
Acetylcholine receptor antibodies	ACHRAb	IgG 1, IgG 3	Disruption of receptor signaling Complement-dependent internalization of receptor
N-methyl-d-aspartate receptor antibodies	NMDARAb	IgG, IgA, IgM	Crosslinking and internalization of receptor
Phospholipase 2 receptor antibodies	PLAR2Ab	IgG	Thickening of capillary wall

### The autoimmunology laboratory and receptor autoimmunity

The measurement of specific pathogenetic autoantibodies using laboratory methods is a challenge for the diagnosis of related autoimmune disease for several reasons:

- The detection of small amounts of pathogenetic autoantibodies in biological fluids, using sensitive immunoassays or bioassays, is the cornerstone for early diagnosis, when the symptoms of the autoimmune disease are not completely clear;
- The measurement of functional antibodies is a key for monitoring the course of related autoimmune diseases, in line with the theoretical dogma (presence/quantity of pathogenetic autoantibodies, presence/intensity of symptoms);
- The role of autoantibody-antigen systems is the hallmark of the successful therapy, not only for remission of the disease, but probably for the definitive health of patients, using recent innovative approaches.

### Graves’ disease and TSH receptor

For historical, pathogenetic, clinical, and diagnostic reasons, Graves’ disease is the paradigm of receptor autoimmune disease. For two centuries GD has been known for its clinical characteristics and the evolution of its knowledge derived from seminal contributions of several authors (Flaiani, Parry, Graves and von Basedow) [11]. The clinical picture of GD is now summarized in the ‘GD triad’, consisting of hyperthyroidism, orbitopathy (Graves’ eye disease) and dermopathy (pre-tibial myxedema) [12]: the multiple clinical forms of GD (from highly localized thyroid disease to systemic extrathyroidal autoimmune disease, involving retro-orbit, skin and bone) [13] are now considered explicable by the variable forms of the TSHR interested by immune activation (monomeric or dimeric), the heterogeneous sites of TSHR expression (thyrocytes, fibroblasts, adipocytes, bone cells, and other cell types) and the multiplicity of biochemical signals and pathways employed by TSHR (G protein dependent or G-protein independent) [14].

TSHR is a member of class A family of G-protein coupled receptors (with the close relatives follitropin and lutropin/choriogonadotropin receptors) that is essential for the function and growth of the thyroid gland and activates different signaling pathways required for thyroid hormones synthesis and release. The receptor structure is constituted by different domains located in different sites of the thyrocyte membrane: the extracellular domain (ECD), the hinge region, and the transmembrane domain (TMD), consisting of extracellular and intracellular loops [15]. After expression on the plasma membrane, the full-length TSHR undergoes cleavage within the hinge region [16]. The loss of a C-peptide leads to an extracellular A subunit (comprising ECD and part of the hinge region), and a B subunit (comprising the remainder of the hinge region, and the TMD): the shed A subunit is the autoantigen initiating and driving the autoimmune response in GD [17].

The full-length TSHR undergoes other complex post-translational processing, including glycosylation, phosphorylation, and multimerization [12, 18]: the multiplicity of TSHR forms probably explains the different phenotypes of GD (thyroid disease only, eye disease only, or ‘complete’ GD) [12].

A unique finding of GD, not present in healthy subjects or in the animal kingdom, is the presence of TSHR autoantibodies (TRAbs), measurable in the majority of patients [15, 19]. TRAbs represent the hallmark of GD. Now we know three varieties of TRAb, present in patients with autoimmune thyroid disease and in TSHR immunized rodents: stimulating (S-TRAbs), blocking (B-TRAbs), and apoptotic (A-TRAbs) and their relative concentrations define the natural history and the clinical picture of disease [17, 20–22].

Due to the progressive improvement of accuracy of bioassay and immunoassay methods, it’s now definitively demonstrated that the laboratory methods are the first choice in current diagnostic approaches, for clinical, analytical, and economic reasons [23–28].

Over the years different assay methods have been proposed and used for TRAb detection/measurement. They are divided in two groups: functional bioassays and non-functional immunoassays. Both bioassays and immunoassays include three different generations based on the evolution over time of assay principles. The third-generation immunoassays include RIA, ELISA, FIA, and CLIA [15, 19, 25].

TRAbs measurement is of central importance also to monitor the successful evolution of GD, in terms of relapse after withdrawal of anti-thyroid drugs therapy, even if this opinion is not fully shared. Recent papers outlined the significance of the predictive value of TRAbs measurement in term of relapse risk, using immunoassay methods and appropriate cutoffs [29–31].

### Myasthenia gravis and acetylcholine receptor

Autoimmune myasthenia gravis is a rare disease, with estimated incidence and prevalence of 0.5–3/100.000 and 7–20/100.000 subjects, respectively [32].

MG is a disorder of neuromuscular junction marked clinically by fatigable muscle weakness and serologically by the presence of autoantibodies, in particular (but not only) against acetylcholine receptor (AChRAb), proven to attack components of the postsynaptic membrane [33].

The autoimmune nature of MG was proven by fundamental works of Patrick [4], Tokya [34], and Lindstrom [35], demonstrating that MG meets all Vitebsky’s diagnostic criteria, in particular the type of autoantigen(s) involved, the related autoantibodies, and the induction of experimental disease in animal models by immunization with purified antigens or passive transfer of human MG antibodies.

Muscle-type nicotine AChR, neurotransmitter member of the ligand-gated ion channels family, is a pentameric molecule located in the in the middle of the post-synaptic membrane, with 5 subunits (α1_2_βδ and ε) in the adult muscle [36, 37]: the acetylcholine binds the receptor at the interface of the α-δ and the α-ε subunits. In the α1 subunit, between the amino acids 67–70 is located the main immunogenic region that plays an important role on the pathogenesis of MG [38].

AChRAbs are present in the 40–90% of patients with MG and allow, together with other antibodies (against MuSK, Lrp4, agrin, etc.), the subclassification of different 9 types of MG, particularly the first 4 types (early onset MG, late onset MG, thymoma-associated MG, and ocular MG) [39]. These autoantibodies induce pathogenicity by three main mechanisms: activation of the classical complement cascade, endocytosis with loss of AChR density, and direct inhibition of AChR binding of ACh or blocking the ACh channel [39].

There are several reliable diagnostic assays to detect autoantibodies against AChR, including RIA and ELISA [40]. In a subgroup of patients, however, AChRAb cannot be detected by these assays.

ACHRAb and other autoantibodies levels appears not to be correlated with disease severity; nonetheless monitoring the levels of MG autoantibodies is likely to provide clinical informations of the disease course in single patients [37].

### Anti-NMDA receptor encephalitis and NMDA receptor

Anti-N-metil-d-aspartate receptor (NMDAR) encephalitis is an inflammatory encephalopathic autoimmune disorder associated with specific autoantibodies against NMDAR that presents a progressive clinical course with the possibility of effective management and favorable outcome.

The NMDAR encephalitis predominantly affects young women. Potential triggers of the disease are tumors, mostly teratomas of the ovary, and much less frequently other tumors. Anti-NMDAR encephalitis is the most common antibody-associated encephalitis [41]. Since its original description [42], there has been a progressive increase in its highlighting, so that now epidemiological data indicate that this disease is responsible for 6–10% of total encephalitides.

Glutamate is the main excitatory neurotransmitter in human brain and targets two different receptor types: the ionotropic receptors (iGluRs) and metabotropic receptors (mGluRs). The iGluRs are important for both synaptic transmission and plasticity, are fundamental in molecular mechanisms of learning and memory, and can be divided in 3 different groups: NMDARs, amino-hydroxy-methyl-isoxazolepropionic acid receptors (AMPARs) and kainate receptors [43]. Unique properties distinguish NMDARs from other iGluRs, particularly the high permeability to calcium ions and the requirement for binding of two coagonists, glutamate and glycine, inducing channel activation.

All NMDARs are heterotetrameric assemblies of different subunits (2 GluN1 and 2 GluN2), which forms a central ion channel for the movements of calcium, sodium, and potassium ions. These subunits share a similar structure that involves four domains: a large extracellular amino-terminal domain (NTD), an agonist binding domain (ABD), a pore transmembrane domain (TMD) and an intracellular domain (CTD) [44].

NMDAR autoantibodies (IgG, IgA, IgM class) are proven to be pathogenic, both in vivo and in vitro ^46^, leading crosslinking and internalization of NMDAR in human cortical neurons, and specific reversible reduction of NMDAR on postsynaptic dendrites. Several different epitopes were identified in ATD, ABD, and CTD [46].

Synaptic dysfunction results in clinical manifestations, such as psychiatric and behavioral symptoms, seizures, motor dysfunctions, memory dysfunction, and speech disorders [41].

Anti-NMDAR autoantibodies can be detected with immunochemistry and cell-based assays (CBA) with fixed or live cells, in cerebro-spinal fluid (CSF) or in serum: in CSF the accuracy of the CBA is absolute (100% accuracy), in serum is lower, with a decrease in sensitivity (87%) [45].

The detection of anti-NMDAR is of importance in monitoring encephalitis, because the levels decrease regardless of outcome [46, 47]. IgG antibodies present high disease specificity, while IgA and IgM may be elevated in healthy individuals and other diseases.

### Idiopathic membranous nephropathy and phospholipase A2 receptor

The idiopathic membranous nephropathy (IMN), the most frequent cause of nephrotic syndrome in adults, is a glomerular autoimmune disease characterized by thickening of the capillary wall, due to subepithelial deposition of immunoglobulin G and complement component C3.

IMN was defined more than 70 years ago by the seminal works of Bell, Jones, and Heymann [48]. After 50 years of clinical and laboratory studies, IMN is now regarded as a podocytopathy dependent on immune deposits of circulating autoantibodies interacting with antigens of the podocyte cell membrane. The main autoantigen involved, but not the unique, is the phospholipase A2 receptor (PLA2R), for the first time highlighted by Beck [8]: this discovery concluded the long odyssey related to the identification of the autoimmune target of the disease [49].

PLA2R is a polypeptide that includes an extracellular domain (ECD), a membrane-spanning, and an intracellular domain. The ECD is composed of a cysteine-rich, a fibronectin type 2-like and eight lectin-like domains [49]. The ECD is folded by disulfide bonds and presents conformational epitopes that are interested by the attack of autoimmune response with anti-PLAR autoantibodies.

IgG4 anti-PLAR antibodies are present in 50–80% of subjects with IMN [50, 51] and virtually absent in secondary forms of IMN and other glomerular diseases [52]. Recently several new commercial immunoassays have been introduced with different assay formats; currently, CBA-IFI, ELISA and MBA-FIA methods are available in clinical practice and the serology quantitative approach is the cornerstone of the diagnosis, differential diagnosis, prognostic evaluation of activity, prediction of remission, and monitoring of post-transplant recurrence of the disease [53–57].

### Other functionally cell surface receptor autoantibodies in autoimmune diseases

The increasing role of receptor autoantibodies is exemplified in other conditions, on which the pathogenesis of clinical symptoms is dependent on the criteria of Rose and Bona [58], which include: the passive transfer of autoantibodies from patients, the reproduction of cellular dysfunction or damage using patient’ sera or immunoglobulins, and the development of main features/symptoms of the disease, after immunization of animals with target antigens.

All previous autoimmune disease fulfill these criteria, but in other conditions the pathogenetic mechanism explains some clinical features, as in the case of systemic sclerosis (autoantibodies against the receptor of platelet-derived growth factor of the fibroblasts, for skin thickening and stiffness, against muscarinic AChR of the visceral smooth muscle, for gastro-intestinal dysmotility, against the type 1 angiotensin II receptor and endothelin-type 1 receptor of endothelial cells, for vasoconstriction) [59], or in the case of dilated cardiomyopathy (autoantibodies against the β_1_-adrenergic receptor of the cardiomyocytes for ventricular dilatation and dysfunction) [60, 61].

Flier syndrome, a rare form of insulin-resistant diabetes characterized mainly by hypoglycemia and presence of blocking or stimulating autoantibodies against insulin receptors, can be counted among the pathologies related to receptor autoimmunity [62, 63].

### Implications for diagnostic use of receptor autoantibodies

Due to the close quantitative correlation between the concentration of anti-receptor autoantibodies and the presence/intensity of symptoms, the measurement of these immunoglobulins has become central to diagnose autoimmune diseases in general, not just in clinically dubious cases. In fact, the diagnostic accuracy of anti-receptor autoantibody tests is generally high, often close to 95–100%, as in the case of Graves’ disease, where a recent meta-analysis showed the high diagnostic power [19] and produced the relocation of the measurement of TRAb as a front-line test in recent U.S. guidelines for autoimmune hyperthyroidism [28].

The measurement of autoantibodies is also relevant for differential diagnosis of autoimmune and non-autoimmune forms with similar symptoms: TRAbs allow to distinguish GD hyperthyroidism from that of toxic multinodular goiter, and PLAR2Abs the nephropathy from IMN from non-IMN [57]; NMDARAbs distinct autoimmune acute from other forms of encephalitis, and AChRAbs are able to classify the various forms of MG, some of which are dependent on other autoantibodies [32, 33, 40].

From the methodological point of view (Table 3), continuous quantitative immunoassay methods of measurement (ELISA, CLIA, MBA, etc.) should be preferred over semi-quantitative ones (IFI, CBA-IFI, etc.), for the capacity granted by the first class of methods to define precisely the reference ranges and decision levels overcoming the measurement uncertainty of dilution methods. In this regard, the availability of quantitative methods for TRAb, AChRAb and PLAR2Ab, but not for NMDARAb (for which only immunohistochemistry and CBA-IFI are available) should be noted: in the latter case, the biomedical industry is called on to produce appropriate efforts to make quantitative methods available for these latter autoantibodies.

**Table 3:** Assay methods for receptor autoantibodies

Autoantibody	Acronym	Method of measurement
TSH receptor antibodies	TRAb	RIA, ELISA, CLIA
Acetylcholine receptor antibodies	ACHRAb	RIA, ELISA
N-metil-d-aspartate receptor antibodies	NMDARAb	Immunochemistry, CBA
Phospholipase 2 receptor antibodies	PLA2RAb	CBA, ELISA, MBA

*RIA* radioimmunoassay, *ELISA* enzyme-linked immunosorbent assay, *CLIA* chemiluminescence immunoassay, *CBA* cell-based assay, *MBA* multiplex bead assay

The quantitative measurement of anti-receptor autoantibodies is equally important in therapy monitoring and prognosis evaluation of autoimmune receptor diseases. If for GD the debate on the threshold values of TRAb as predictors of remission/relapse remains open, the importance of ascertaining seronegative patients after withdrawal of therapy is not in question, because they have a better prognosis than those who are seronegative at various levels of concentration [29]. This last statement is certainly also demonstrated for PLA2RAb in IMN [55, 56], but not yet for MG or NMDAR encephalitis.

### Implications for therapeutic use of receptor peptides and autoantibodies

Recently, starting from the knowledge of the molecular structure of TSHR, promising results have been highlighted by the use of an antigen-specific immunotherapy of Graves’ disease and Graves’ orbitopathy, using small amounts of synthetic peptides derived from the TSH receptor, that mimic naturally processed CD+T cell-epitopes [64, 65]. This first demonstration of the effectiveness of a specific therapy, which induces immunotolerance for Graves’ endocrinopathy [13], paves the way for new therapeutic approaches in many, if not all, autoimmune receptor diseases.

## Conclusions

The knowledge of a group of autoimmune diseases with common findings related to pathogenic, diagnostic and therapeutic mechanisms is critical for clinical and laboratory autoimmunologists with the goals of standardization/harmonization of laboratory tests and therapeutic solutions for receptor human pathologies.

## Data Availability

Not applicable.
